# Geriatric Migraine, Geroscience, and Sustainable Development Goals: Bridging Clinical Complexity and Public Health Priorities

**DOI:** 10.3390/jcm15083088

**Published:** 2026-04-17

**Authors:** Claudio Tana, Michalis Kodounis, Raffaele Ornello, Bianca Raffaelli, Roberta Messina, William Wells-Gatnik, Marta Waliszewska-Prosół, Simona Sacco, Dilara Onan, Paolo Martelletti

**Affiliations:** 1 Internal Medicine Unit, Eastern Hospital, Azienda Sanitaria Locale of Taranto, 74121 Taranto, Italy; 2 First Neurology Department, Eginitio Hospital, School of Medicine, National & Kapodistrian University of Athens, 11528 Athens, Greece; 3 Department of Biotechnological and Applied Clinical Sciences, University of L’Aquila, 67100 L’Aquila, Italy; 4 Department of Neurolog, Charité-Universitätsmedizin Berlin, 10117 Berlin, Germany; 5 Neurology Unit, Division of Neuroscience, IRCCS San Raffaele Scientific Institute, 20122 Milan, Italy; 6 Neurology Unit, Vita-Salute San Raffaele University, 20132 Milan, Italy; 7 Department of Neurology, Texas Tech University Health Sciences Center El Paso, El Paso, TX 79905, USA; 8 Department of Neurology, Wroclaw Medical University, 50-367 Wrocław, Poland; 9 Department of Physiotherapy and Rehabilitation, Faculty of Health Sciences, Yozgat Bozok University, 66100 Yozgat, Türkiye; 10 School of Health, Unitelma Sapienza University of Rome, 00161 Rome, Italy; 11 Tongji Medical College, Huazhong University of Science and Technology, Wuhan 430030, China

**Keywords:** migraine, older adults, frailty, pain, quality of life, polypharmacy, CGRP

## Abstract

**Background:** Migraine in older adults represents an increasingly relevant yet underrecognized clinical challenge in aging societies, where multimorbidity, frailty, and polypharmacy complicate both diagnosis and management. Although traditionally considered a disorder of younger individuals, migraine frequently persists or presents after the age of 60 with atypical features, contributing to diagnostic uncertainty. **Methods:** This narrative review, conducted in accordance with the SANRA principles, aims to provide a comprehensive overview of the epidemiology, clinical presentation, pathophysiology, and management of migraine in older adults, with particular emphasis on age-related complexities, therapeutic challenges, and unmet clinical needs. **Results:** Migraine in this population often presents with atypical or misleading features, such as aura without headache, vestibular symptoms, or overlap with cerebrovascular conditions, leading to delayed or incorrect diagnoses. The burden of disease is substantial, affecting physical function, mobility, cognition, emotional well-being, and social participation, and is further amplified by comorbid conditions including cardiovascular and metabolic disorders, mood disturbances, and chronic pain syndromes. Aging-related neurobiological changes, such as impaired pain modulation, endothelial dysfunction, and neuroinflammation, may influence disease expression and treatment response. Therapeutic management is challenged by contraindications, increased susceptibility to adverse drug effects, and the complexity of polypharmacy, highlighting the importance of individualized and non-pharmacological approaches. **Conclusions:** Migraine in older adults is a significant but often overlooked contributor to disability and reduced quality of life. Improved recognition of its unique clinical features and age-specific vulnerabilities is essential to optimize patient-centered care. Future research should prioritize the inclusion of older populations and the development of tailored, safe, and effective management strategies.

## 1. Introduction

The global demographic landscape is undergoing a profound transformation, characterized by an unprecedented increase in the older adult population. As the World Health Organization and the United Nations champion the Decade of Healthy Ageing 2021–2030 [1], a critical yet often overlooked clinical challenge demands attention: enhancing healthcare management and administration, particularly in geriatric care [1,2]. This gap is especially evident in Low- and Middle-Income Countries (LMICs), where the WHO’s historical focus has long been dominated by Neglected Tropical Diseases (NTDs). It is only in recent years that the WHO/UN agenda has been expanded to include Non-Communicable Diseases [1].

Beyond epidemiological transitions, aging is associated with a progressive accumulation of functional impairments affecting mobility, cognition, and psychosocial well-being, which collectively contribute to increased disability and healthcare needs [2]. In this context, chronic conditions may act as amplifiers of pre-existing vulnerabilities rather than isolated diseases. Accordingly, their clinical relevance should be interpreted within a broader geriatric and public health framework, linking disease burden to global priorities such as healthy aging and disability reduction within the Sustainable Development Goals (SDGs) [3]. In particular, migraine in older adults can be directly mapped to key SDG targets, including SDG 3 (Good Health and Well-being), through its contribution to disability and reduced quality of life; SDG 10 (Reduced Inequalities), given disparities in access to diagnosis and treatment; and SDG 12 (Responsible Consumption), in relation to polypharmacy and medication overuse. This framing highlights migraine as not only a clinical condition but also a relevant public health priority within global aging strategies [3]. While migraine has traditionally been perceived as a disorder of young and middle-aged adults, its persistence and manifestation in later life carry indeed significant implications for quality of life and disease burden, warranting careful examination. Migraine prevalence in older adults remains substantial, despite a general decline after the fifth or sixth decade of life. Current evidence indicates that approximately 7% of women and 3% of men over 65 years continue to experience migraine, with prevalence rates of 6% in women and 3% in men persisting even beyond 80 years of age [4,5]. The Global Burden of Disease Study 2023 further underscores the significance of headache disorders, reporting that migraine alone accounted for 487.5 years lived with disability (YLDs) per 100,000 population globally, with rates more than twice as high in females as in males [6,7]. These figures challenge the misconception that migraine naturally remits with age and highlight the need for targeted attention to this population. The clinical presentation of migraine in older adults often deviates from the classical patterns observed in younger populations, yet it remains poorly characterized by robust epidemiological data [8]. Research has identified several age-related modifications, including reduced attack frequency, decreased pain intensity, and the emergence of late-onset aura phenomena [8]. The diagnostic complexity of migraine in older age is compounded by age-related comorbidities and an increased risk of secondary headache disorders that may mimic migraine [9,10]. Polypharmacy, physiological changes affecting drug clearance, and potential cognitive or mobility impairments further complicate both assessment and treatment in this population [11]. Standard of Care (SoC) options, while commonly used, are frequently associated with adverse effects that limit their tolerability in older adults [12,13,14]. The impact of migraine on quality of life among older adults extends beyond pain and disability. A study of underserved older African American adults demonstrated that migraine was significantly associated with higher healthcare utilization, including emergency department admissions and increased medication use. This was alongside lower physical and mental quality of life, greater depressive symptoms, higher pain levels, sleep disorders, and increased disability [15]. These findings illuminate the multifaceted burden that migraine imposes on well-being in later life.

Despite this growing clinical need, older adults remain strikingly underrepresented in migraine research. Most randomized controlled trials of preventive therapies, including onabotulinumtoxinA, greater occipital nerve blocks, and anti-calcitonin gene-related peptide monoclonal antibodies, have either excluded patients over 65 years or included only small, unanalyzed proportions. This gap in knowledge is particularly concerning given the projected increase in the global population aged 65 years and older, which is projected to reach approximately 18.51% among OECD (Organisation for Economic Co-operation and Development) countries by 2024 (up from 9.32% in 1970) [16]. Understanding these factors is essential for developing effective, evidence-based strategies to improve outcomes and preserve quality of life for older adults living with migraine. In this context, this review aims to provide a comprehensive overview of migraine in older populations, addressing its epidemiology, clinical presentation, comorbidities, and management challenges within a geroscience and public health framework. By integrating clinical complexity with broader health system implications, this work seeks to highlight current gaps in knowledge and inform future research and tailored therapeutic approaches for this growing and vulnerable population.

## 2. Methods

This narrative review was conducted to summarize current evidence on migraine in older adults, with a focus on epidemiology, clinical features, pathophysiology, comorbidities, and management strategies. The review was developed in accordance with the SANRA (Scale for the Assessment of Narrative Review Articles) guidelines, which provide a structured framework to ensure quality and transparency in narrative reviews.

A comprehensive literature search was performed using the PubMed/MEDLINE, Scopus, and Web of Science databases up to February 2026. The following keywords and Medical Subject Headings (MeSH) terms were used: “migraine”, “older adults”, “elderly”, “aging”, “geriatric”, “comorbidity”, “polypharmacy”, “quality of life”, “calcitonin gene-related peptide, CGRP”, and “treatment”. These terms were combined using Boolean operators (AND/OR).

Studies were selected based on their relevance to the topic, with particular attention paid to those involving individuals aged ≥60 years or providing age-specific analyses. Priority was given to original research articles, randomized controlled trials, observational studies, and relevant reviews published in English. Case reports and editorials without original data were considered only when deemed informative, while studies not specifically addressing migraine or lacking meaningful insights into older populations were generally not prioritized. Overall, article selection was guided by methodological soundness and clinical relevance to the aims of the review. In addition, where appropriate, the level of evidence was qualitatively categorized as: (i) evidence derived from studies specifically involving older populations, (ii) evidence extrapolated from the general adult migraine literature, and (iii) hypothesis-generating or mechanistic interpretations.

## 3. Epidemiology of Migraine in Older Adults

Migraine is a chronic neurological disorder that reaches its highest prevalence in early and middle life [6]; however, it may persist into later life, may not improve after transitions such as postmenopause, and may rarely recur at an advanced age [17]. While the female-to-male ratio of migraine in young and adult individuals is approximately 3:1 [18,19], among individuals over 65 years of age, although overall prevalence decreases, a significant female predominance persists, with ratios generally reported in the range of 2–3:1 [20,21]. Although the prevalence of active migraine (defined as having experienced an attack within the last year) is generally lower in older adults compared to younger adults, migraine remains a significant health problem clinically in the elderly population [21]. Studies have reported an annual migraine prevalence of approximately 10% in older adults [17,22]. It has also been reported that chronic forms of migraine can become more persistent with age, and migraine is one of the most common primary headaches in older adults after tension-type headache [6].

Large-scale global burden analyses have shown that the worldwide prevalence of migraine increased from 732.56 million in 1990 to 1.16 billion in 2021, representing a 58.15% increase [23]. However, this rise in prevalence can largely be attributed to global population growth [23]. Furthermore, the burden measured in disability-adjusted life years (DALYs) also increased from 27.41 million in 1990 to 43.38 million in 2021, a 58.27% increase [24]. A community-based study in elderly individuals reported that most patients with active migraine had fewer than 9 headache days per month; that migraine was associated with depression, poor sleep quality and low physical activity levels; and that the disease burden should be assessed more through functional impact rather than continuity [21]. Although there is a decreasing trend in the prevalence, incidence, and DALYs of migraine among age groups, the functional loss and impaired quality of life caused by migraine in the elderly population continue to constitute a significant burden on public health [24]. One study reported that migraine was the most frequently reported type of headache (13.8%) among individuals over 65 who sought medical help due to cognitive and memory problems, and that this was a condition requiring attention in treatment [25]. These findings suggest that the overall burden of migraine is projected to increase through 2050, that migraine is not solely a disorder of young adulthood, and that the burden on health systems will continue in the context of demographic aging [24,26,27].

Factors influencing the epidemiology of migraine in elderly individuals include female sex, a history of migraine earlier in life, comorbid psychiatric disorders (presence of depression, anxiety), sleep disorders, cardiovascular (CV) comorbidities, and medication use profile [21]. Furthermore, some studies have shown an increased incidence of new-onset headaches in elderly individuals, and although rare, late-onset migraine has also been described [28,29]. This suggests that late-life migraine may represent an extension of the primary migraine course; however, secondary causes of headache such as vascular diseases or giant cell arteritis are more prevalent in this age group, underscoring the need for careful epidemiological assessment and thorough clinical evaluation [30].

## 4. Clinical Presentation and Diagnostic Challenges

The clinical presentation of migraine in older adults may differ from that observed in younger individuals. Typical changes include reduced attack severity, more frequent reports of bilateral headache, less prominent sensory symptoms such as photophobia and phonophobia, a higher frequency of reported aura phenomena, and an increased occurrence of autonomic symptoms, including tachycardia and facial flushing [5,31]. Collectively, these features may complicate the diagnosis of cases that do not fully meet the criteria of the International Classification of Headache Disorders, third edition (ICHD-3) [31].

Although migraine can be diagnosed clinically according to the criteria of the ICHD-3, the first onset or diagnosis of migraine after the age of 50 necessitates a thorough differential diagnostic evaluation for secondary headaches (such as stroke or intracranial tumors) [9]. In addition, the presence of comorbidities and polypharmacy, as well as patients’ reduced ability to clearly report their clinical history due to age-related cognitive changes, further complicate the diagnostic process and require careful physician assessment [5,9,30].

One of the most significant diagnostic challenges in elderly patients presenting to clinics with headache complaints is ruling out secondary headaches [29,32]. Potentially life-threatening causes, including cerebrovascular events, intracranial neoplasms, subdural hematoma, and giant cell arteritis, must be carefully considered [5]. An additional challenge is the high prevalence of comorbid conditions in older adults [30]. Disorders such as hypertension, prior cerebrovascular events, ischemic heart disease, atrial fibrillation, and depression may obscure typical migraine features, thereby reinforcing concern for secondary etiologies and complicating the diagnostic assessment [30].

Polypharmacy can also restrict the diagnostic process and treatment options for migraine due to the increase in comorbidities associated with advanced age, or it can complicate the assessment of migraine symptoms and treatment options due to the increased risk of headaches resulting from excessive analgesic use [30]. The atypical nature of migraine symptoms can also make diagnosis difficult in older adults. Individuals may describe headaches as being less severe but more frequent, and they may not report distinctive symptoms such as vomiting, nausea, and photophobia/phonophobia to the physician, considering them to be a natural part of aging [5,31]. On the other hand, deficiencies in describing symptoms may also occur in cases of decreased memory and cognitive impairment, such as dementia, that accompany age [33].

Given these challenges, adequate time should be devoted to obtaining a detailed and structured medical history. Systemic conditions, particularly neurological and CV diseases, should be carefully explored using a multidisciplinary approach. Red flags must be actively sought, with prompt use of appropriate laboratory investigations and neuroimaging when indicated. In addition, assessment of functional status, including independence and activities of daily living, is essential for the accurate diagnosis of migraine in older adults and for minimizing diagnostic risk [5,30].

## 5. Comorbidities and Differential Diagnosis

Older adults with migraine are more likely to have comorbid conditions than their younger counterparts. Data from the American Migraine Prevalence and Prevention (AMPP) study showed that the prevalence of CV disease increased from 3.4% in subjects aged 22–39 years to 22.3% in those aged ≥60 years, while the prevalence of CV procedures increased from 1.1% to 8.8% [34,35]. CV risk factors and events increase substantially with advancing age, and this trend has important implications for migraine management in older adults. Conditions such as hypertension, hyperlipidemia, diabetes, and atherosclerotic disease become more prevalent over time, contributing to higher rates of stroke, myocardial infarction (MI), and other vascular complications. In parallel, a systematic review of 64 studies (97,846 patients) identified multiple risk factors also associated with headache-related disability, according to the Global Burden of Disease (GBD) framework. The most consistently reported were metabolic factors such as high body mass index (BMI), elevated fasting plasma glucose, and increased LDL cholesterol, and behavioral factors, including physical inactivity, unhealthy diet, smoking, and alcohol consumption [34,35,36]. In this context, migraine with aura represents an additional independent risk factor for vascular morbidity and mortality, further complicating clinical decision-making [36]. The coexistence of these vascular conditions often necessitates careful diagnostic evaluation and may limit the use of commonly prescribed acute and preventive migraine therapies, particularly those with vasoconstrictive properties [5]. Psychiatric comorbidities, especially depression and anxiety, are also highly prevalent in older individuals with migraine and contribute significantly to disease burden [37,38]. A comparative study showed higher prevalence of depression and poorer sleep quality in elderly migraine patients compared with younger migraine patients [39]. These conditions may promote maladaptive coping behaviors, including frequent use of analgesic medications, thereby increasing the risk of medication-overuse headache (MOH) [37]. Concomitant use of multiple medications represents an additional challenge in older adults. The need to treat coexisting chronic conditions increases the risk of drug interactions, adverse effects, and poor adherence, often limiting therapeutic options for migraine and contributing to symptom burden [5].

With advancing age, the differential diagnosis of migraine is more difficult than in the young given the different manifestations of the disease. Migraine attacks can become more frequent and less severe in older compared with younger adults [38]. Additionally, compared with younger subjects, elderly individuals have fewer symptoms typical of migraine, such as the unilateral location and nausea or vomiting [39,40]. In the population of older adults, given the diagnostic challenges for migraine, mimics and chameleons of the disease should be systematically sought. Migraine with aura can be mimicked by harmful secondary headache disorders, such as giant cell arteritis, which can lead to blindness if left untreated [41]. The symptoms of migraine with aura can also be mimicked by cerebrovascular disorders such as subarachnoid hemorrhage, arteriovenous malformations, and arterial dissections; occipital lobe seizures and brain tumors such as meningiomas can also lead to visual symptoms similar to aura [42]. Other conditions typical of older age that need to be ruled out when encountering a migraine-like headache disorder include hypothyroidism, cardiac cephalalgia, glaucoma, carotid-cavernous fistulas, and obstructive sleep apneas [17]. For those reasons, a change in migraine frequency or characteristics in elderly individuals should prompt clinicians to perform additional diagnostic exams [43] including cervical spine MRI, Doppler sonography of the neck vessels, and laboratory exams such as erythrosedimentation rate.

Together with mimics, the clinical investigation of migraine should consider chameleons, i.e., clinical manifestations related to migraine that resemble other disorders. Older age is associated with an increased occurrence of aura and decreased occurrence of headache compared with younger age [44,45]. Thus, in older individuals, aura without headache may occur and can be challenging to distinguish from transient ischemic attack (TIA), particularly in patients with vascular risk factors [39]. There are clinical rules to distinguish migraine with aura from TIA [46]. Overall, the new onset of headache after 50 years of age is a “red flag” for secondary headache disorders even in the presence of clinical symptoms typical of migraine [5].

## 6. Impact on Quality of Life and Functional Status

Data specifically addressing the impact of migraine on health-related quality of life (HRQoL) and functional status in older adults remain limited. Nevertheless, converging evidence from studies including middle-aged and older individuals, together with consideration of age-specific health challenges, permits meaningful conclusions regarding the substantial burden of migraine in this age group. Overall, migraine exerts a marked negative effect on physical, psychological, and social dimensions of quality of life in older adults, with important implications for functional independence [5].

Population-based studies show that older adults with migraine report significantly lower HRQoL compared with age-matched individuals without migraine. In a large Canadian population-based cohort of middle-aged and older adults between 45 and 85 years of age, individuals with migraine rated their physical, mental, and oral health significantly worse than those without migraine [47]. These findings are consistent with broader evidence demonstrating that migraine negatively affects all domains of quality of life, including physical functioning, role limitations, bodily pain, general health perceptions, vitality, social functioning, and emotional well-being [6]. The negative impact of migraine on HRQoL persists into older age and remains clinically relevant despite age-related changes in migraine phenotype.

Older adults with migraine report significantly higher levels of activity limitation and functional impairment than those without migraine, with pain and discomfort interfering with daily activities [47,48]. Although migraine attacks in older adults may be individually less severe and less disabling than in younger individuals, they nonetheless result in a sustained and cumulative burden. This is particularly relevant given the relatively high proportion of chronic migraine among older individuals who continue to experience migraine, estimated at approximately 12% in men and 8% in women aged 60 years and older [35]. Increased attack frequency translates into a greater proportion of time spent in a symptomatic or recovery state, amplifying the impact on daily functioning, and contributing to reduced independence. Evidence from adult migraine populations indicates a clear dose–response relationship between headache frequency and disability: individuals with high-frequency episodic migraine (8–14 monthly headache days) show severe disability in 42–44% of cases, compared with 11–15% among those with low-frequency episodic migraine (0–7 monthly headache days), as measured by the Migraine Disability Assessment (MIDAS). Disability is even more pronounced in chronic migraine, where 58–66% of individuals report severe impairment [6,49]. Work productivity and activity impairment increase in parallel, with overall activity impairment rising from approximately 29–36% in low-frequency episodic migraine to 52–54% in chronic migraine [49]. Given the higher attack frequency observed in many older adults with persistent migraine, these findings strongly suggest a clinically meaningful impact on functional capacity in this population.

Beyond general disability, migraine is associated with specific impairments in balance and mobility that are highly relevant to aging-related vulnerability. Objective balance assessments using the Sensory Organization Test show lower composite scores in all migraine subgroups compared with controls, with particularly pronounced deficits in individuals who experience migraine with aura and chronic migraine [50]. These impairments occur even in the absence of overt otoneurological abnormalities and are accompanied by higher rates of falls during balance testing, especially among patients with migraine with aura [50]. Balance dysfunction correlates with migraine frequency, fear of falling, dizziness-related disability, and kinesiophobia, creating a complex interaction between physical and psychological factors that further limits mobility and participation. In older adults, such impairments are of particular concern given their established association with falls, injuries, and loss of independence.

Functional limitations frequently translate into increased dependence on caregivers. Among individuals with frequent migraine (≥4 monthly migraine days), approximately 70% rely on family members, friends, or others for assistance with daily activities, receiving help for a median of 9 days over a 3-month period [51]. This dependence is even greater in chronic migraine, where assistance is required for a median of 10 days per 3 months. Importantly, the need for support extends beyond the headache phase itself, encompassing the premonitory, aura, and postdrome phases, underscoring the pervasive nature of migraine-related functional impairment. In older adults, who may already rely on external assistance because of comorbidities, frailty, or sensory and mobility limitations, migraine-related disability is therefore likely to further increase dependence and accelerate loss of functional independence.

Psychological and emotional well-being are also substantially affected. Older adults with migraine exhibit higher rates of anxiety, depression, and mood disorders than age-matched controls, which are associated with worse quality of life, increased disability, and greater pain chronification [47]. Notably, the association between migraine and depression appears stronger in individuals older than 60 years than in younger populations [5]. A comparative study showed higher prevalence of depression and poorer sleep quality in elderly migraine patients compared with younger migraine patients [48]. These psychiatric comorbidities substantially worsen HRQoL and contribute independently to disability and reduced functioning.

Cognitive and perceptual dimensions of quality of life are also affected. Older adults with migraine more frequently perceive cognitive decline and report greater concern about memory problems compared with individuals without migraine [47]. More than twice as many individuals with migraine report having been told by a physician that they have memory problems, despite objective testing revealing minimal differences or even slightly better performance [47]. This discrepancy highlights the relevance of subjective cognitive complaints, which may adversely affect well-being, confidence, and participation in daily activities irrespective of objective cognitive performance.

Social participation and role functioning are further compromised. Migraine interferes with family relationships, social engagement, and the ability to maintain professional and social roles [5]. Qualitative studies describe experiences of isolation, frustration, guilt, and internalized stigma, as well as fear-driven avoidance behaviors and pain catastrophizing that limit engagement in daily life. Sleep disturbances and anticipatory anxiety are highly prevalent, with 87% of individuals with frequent migraine reporting sleep problems and 41% expressing severe fear of the next attack, rising to 48% among those with multiple preventive treatment failures [51].

Migraine in older adults is often characterized by the co-existence of MOH, which is associated with increased headache frequency and poorer quality of life [48]. In addition, common age-related comorbidities such as CV disease, hypertension, and chronic pain syndromes complicate management and may limit therapeutic options, increasing the risk of polypharmacy and reduce quality of life [5].

In summary, migraine in older adults is associated with a profound and multidimensional reduction in quality of life and functional status. Even in the context of limited age-specific data, evidence from mixed-age populations and consideration of aging-related vulnerabilities strongly indicate that migraine contributes to physical disability, impaired mobility and balance, psychological distress, social restriction, and caregiver dependence in this population.

## 7. Disease Burden and Healthcare Utilization

As previously discussed, migraine prevalence declines progressively after midlife, becoming relatively low among individuals aged ≥60 years and decreasing further beyond age 70 [44]. However, this decline does not correspond to a proportional reduction in the individual burden of the disease, which continues to impose significant health, economic, and healthcare system challenges in later life [24].

At the population level, global data highlight the persistent and growing burden of migraine across the lifespan. In recent decades, migraine-related burden increased by nearly 60%, largely driven by population growth and demographic aging. As populations continue to age worldwide, this trend is expected to persist. Although age-standardized prevalence rates have remained relatively stable, the absolute number of older individuals living with migraine is steadily rising. Consequently, migraine is likely to remain a major public health challenge in aging societies [6].

As previously discussed, migraine in older adults is often characterized by a more complex clinical profile, including higher rates of chronic migraine, MOH, and multimorbidity. Tertiary care studies indicate that migraine frequently persists into later life in a subset of older adults, commonly presenting as chronic forms associated with substantial clinical burden [52]. The proportion of chronic migraine among those who remain affected increases with age, reaching approximately 12% in men and 8% in women aged 60 years or older, although estimates vary according to diagnostic criteria and study methodology. Chronic migraine is particularly burdensome in this population, as it is associated with greater disability, increased healthcare utilization, and higher treatment costs [5].

Healthcare utilization in older individuals with migraine is frequently increased, reflecting greater diagnostic complexity, multimorbidity, and therapeutic constraints. In later life, diagnostic evaluation aimed at excluding secondary causes of headache, including neuroimaging and specialist consultation, is more frequently required, often leading older adults to access emergency departments as well as radiology and specialist services, with consequent increases in healthcare utilization and expenditure [53]. Beyond diagnostic considerations, migraine in older adults contributes to substantial healthcare resource utilization. Population-based and register-based studies indicate higher rates of outpatient visits, prescription medication use, and hospitalizations among older individuals with migraine compared with age-matched controls, largely driven by comorbid conditions and greater clinical complexity [54,55]. Together, these findings suggest that healthcare use in this population reflects not only disease persistence but also the cumulative impact of age-related multimorbidity and diagnostic uncertainty.

**Figure 1:**
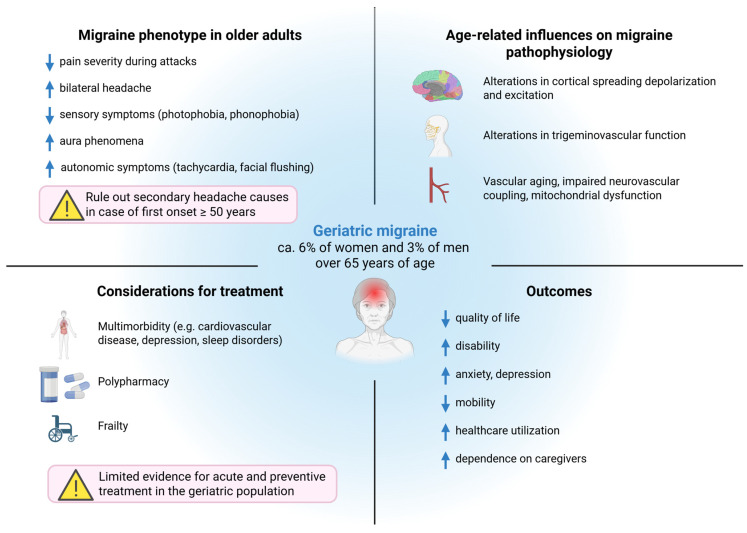
Geriatric migraine: clinical phenotype, pathophysiological mechanisms, treatment considerations, and outcomes in older adults. Aging is associated with changes in migraine presentation, including reduced pain intensity and sensory symptoms, alongside increased diagnostic complexity. These clinical features, together with multimorbidity, polypharmacy, and frailty, contribute to greater disability, reduced quality of life, and increased healthcare utilization. Down arrow: Reduced; Up arrow: increased.

Figure 1 provides an overview of geriatric migraine, highlighting age-related modifications in clinical phenotype, underlying biological mechanisms, and the complex interplay between multimorbidity, treatment challenges, and outcomes.

## 8. Pathophysiological Considerations of Migraine in Aging

Aging influences multiple systems associated with the pathophysiology of migraine, including cortical excitability, neurovascular–nociceptive coupling, and hormonal modulation [5]. As such, these age-related factors appear to influence the evolving migraine phenotype as seen in older adults. Therefore, a brief review discussing the cellular, vascular, and molecular mechanisms underlying the decreased frequency and intensity of migraine episodes amongst older adults is necessary to develop a further understanding of migraine in this population. However, it should be noted that several of the mechanisms discussed are derived from experimental models or studies not specifically focused on older populations; therefore, their application to older adults should be interpreted with caution and considered largely hypothesis-generating.

### 8.1. Age-Related Alterations in Cortical Spreading Depolarization and Excitation

Cortical spreading depolarization (CSD), a well-defined electrophysiological substrate of migraine aura, persists in older adults [5]. However, for older adults with migraine with aura, it appears that increased cortical pain thresholds, diminished excitatory transmission, and altered network connectivity may collectively dampen the clinical expression of CSD and limit CSD–nociceptive coupling [5], a phenomenon that is likely independent of structural brain changes commonly seen with aging.

Aging has been associated with suppressed dendritic arborization, synaptic density, and excitatory neurotransmitter release, which attenuate cortical responsiveness to excitatory triggers [5,20]. Concurrently, enhanced inhibitory GABAergic networks limit signal conduction and excitatory propagation, increasing the threshold required to initiate CSD amongst older adults [20]. These age-related synaptic changes likely define why increased stimuli are required to evoke CSD and therefore acute migraine episodes in older adults with migraine with aura.

Age-related inflammation and functional alteration to microglia have also been demonstrated to modulate CSD to nociceptive signaling [17,20]. Dystrophic microglia, as seen in older adults, exhibit impaired motility and cytokine responsiveness, reducing the amplification and hence propagation of typical nociceptive signaling that follows CSD [17]. Consequently, older adults may continue to demonstrate episodes of electrophysiological CSD initiation in the absence or significant attenuation of acute headache or migraine pain. 

### 8.2. Age-Related Decline in Trigeminovascular Function

The role of neuropeptide and nociceptive signaling within the trigeminovascular system (TVS) in the pathogenesis and pathophysiology of migraine continues to represent an area of intense interest. Signaling cascades involving neuropeptides such as CGRP, substance P, and pituitary adenylate cyclase-activating polypeptide-38 (PACAP38) continue to be investigated as leading explanations of migraine pathogenesis [4].

Early animal studies have reported an age-related functional decline in neuropeptide concentrations within the TVS. With increased age, neuropeptides including CGRP and substance P are hypothesized to undergo decreased expression, impaired axonal transport, and diminished release from sensory nerve terminals [5,20]. A similar phenomenon, although poorly studied, may be predicted in alternate migraine-related neuropeptide signaling pathways within the TVS, such as in PACAP-mediated signaling pathways. 

### 8.3. Vascular Aging, Impaired Neurovascular Coupling, and Mitochondrial Dysfunction

With increased age, arteriolar walls possess a reduced concentration of elastin and smooth muscle, which is often replaced with increased collagen. As a result, vessel distensibility is reduced and may impair signaling from the mechanical and ionic stimuli necessary for perivascular nociceptor activation [5]. As decreased vascular capacitance correlates with both reduced migraine frequency and severity, it is plausible that impaired cerebrovascular responsiveness may impair the ability of CSD to trigger trigeminocervical nociceptors.

Endothelial dysfunction may further reduce perivascular nociception and support neurovascular-related decoupling. Endothelial dysfunction is attributed to pathways that lead to impaired nitric oxide-mediated vasodilation, dysregulated prostacyclin signaling, and diminished vascular response to vasodilatory stimuli [5]. These factors may reduce oxygen and metabolic support to cortical and trigeminal circuits, further impairing excitatory transmission and neuropeptide release and causing integrated attenuation of migraine in older adults and comorbid cerebrovascular disease [5,17]. However, it should be noted that there remains a paucity of data supporting these speculative assertions. 

### 8.4. Hormonal Modulation and Sex-Specific Effects

Hormonal influences have been shown to significantly contribute to the pathophysiology of migraine and vary greatly across the course of life. In women, fluctuations in estrogen signaling strongly correlate with migraine activity, as demonstrated by epidemiologic data showing exacerbations of migraine activity during menstruation, in the postpartum period, and throughout perimenopause [4,5]. However, a decrease in hormonal cyclicity and stabilization of estrogen and progesterone levels after menopause has been associated with decreased migraine frequency [4].

Sex-specific differences in CSD susceptibility further illustrate the role of hormonal modulation in the pathophysiology of migraine. Estradiol has been shown to increase CSD initiation [24], whereas testosterone signaling appears to provide protective effects against nociception and conditions associated with chronic pain, likely contributing to the female predominance attributed to migraine [24].

With increasing age, endogenous testosterone production declines significantly. However, migraine frequency and intensity do not appear to increase among older men. This pattern may reflect a concurrent reduction in estrogen signaling in older adults, potentially offsetting the pro-nociceptive effects associated with declining testosterone levels.

In line with this hormonal convergence, the female predominance in migraine prevalence appears to attenuate with advancing age, likely due to a progressive convergence in sex-related hormonal signaling [24].

## 9. Therapeutic Approaches and Their Safety in Geriatric Migraine

From a geroscience perspective, geriatric migraine management differs substantially from that in younger adults due to age-related pharmacokinetic/pharmacodynamic changes, along with decreased renal and hepatic clearance, changes in body composition, altered drug receptor sensitivity and polypharmacy [56,57]. In addition, individuals aged 65 years and older, especially those with multiple comorbidities, are frequently excluded from clinical trials, both for conventional and non-conventional treatments, resulting in limited evidence to guide treatment decisions in this population [58,59].

### 9.1. Acute Treatments and Their Safety

#### 9.1.1. Simple Analgesic Drugs and Nonsteroidal Anti-Inflammatory Drugs

Acetaminophen (paracetamol) is a frequently preferred, safe drug for the acute symptomatic treatment of migraine attacks in older adults [60]. However, dose adjustment and monitoring of liver function are important in older adults due to pharmacokinetic changes, concomitant drug use, and conditions such as liver disease; furthermore, frequent analgesic use may increase the risk of drug overdose and drug-induced headache. However, acetaminophen is preferred over nonsteroidal anti-inflammatory drugs (NSAIDs) as it has fewer side effects [60].

NSAIDs are among the first-line treatment options for acute migraine attacks and are effective in mild to moderate attacks [61]. NSAIDs, such as ibuprofen, aspirin, naproxen, and diclofenac potassium, which are recommended in international guidelines and have good evidence of efficacy, are generally used in the treatment of patients of all ages [62]. However, long-term or frequent use of NSAIDs in older adults increases the risk of gastrointestinal bleeding, increased arterial pressure, impaired renal function, and CV adverse events [61]. Therefore, NSAID use in older adults should be preferred under medical supervision, for a limited duration, and at the lowest effective dose; contraindications such as a history of peptic ulcer/bleeding, chronic kidney disease, heart failure, or anticoagulation should be carefully evaluated [61]. In addition, drug interactions and concomitant use with anticoagulants/antiplatelets require particular attention in the presence of polypharmacy. General guidelines and reviews recommend considering alternative pharmacological approaches in older adults with NSAID contraindications [5,57].

#### 9.1.2. Triptans and Ditans

Evidence on triptans regarding their efficacy and safety in older adults is still limited, as individuals over 65 years of age and those with comorbidities are generally excluded from randomized controlled trials. Current studies report a low incidence of acute vascular events in triptan users over 65 years of age, but a small increase in cerebral events such as stroke [63]. Additionally, caution may be warranted regarding dyspnea, the most commonly reported adverse effect of sumatriptan, and nausea, vomiting, and terminal ileitis associated with zolmitriptan, rizatriptan, and naratriptan [64].

Ditans (lasmitidan) are a class of drugs that do not exhibit vasoconstrictor effects. This group can be preferred in cases where triptans have an inadequate response or if patients have CV diseases. Lasmiditan is tolerable in older adults, but caution is required due to its potential side effects such as sedation, dizziness, and risk of falls [65].

#### 9.1.3. Use of Gepants in Acute Attacks

Gepants (ubrogepant, rimegepant, atogepant) are CGRP antagonists, a new class of drugs that do not exhibit vasoconstrictive effects, do not have CV contraindications like NSAIDs and triptans, and are well-tolerated. However, evidence is limited for their use in migraine in older adults [66,67,68]. Because gepants are metabolized by CYP3A4, the US Food and Drug Administration recommends caution regarding potential drug interactions [69].

#### 9.1.4. Opioids and Barbiturates

Opioids and barbiturate drugs are not recommended in the treatment of acute migraine in older adults due to their low efficacy and risk of addiction and side effects [70,71]. Table 1 shows the main acute treatments for migraine in older adults.

### 9.2. Prophylactic Treatments and Their Safety

The patient’s body composition, accompanying comorbidities, polypharmacy, drug tolerance, individual characteristics, and preferences should be taken into account in the choice and dose adjustment of drugs, and patients should be closely monitored. In general, some drugs targeting CGRP pathways are recommended for safety after investigation up to the age of 80, and OnabotulinumtoxinA is also recommended in elderly individuals with chronic migraine due to its minimal systemic side effects [72]. The main preventive treatments are listed in Table 2.

#### 9.2.1. Beta-Blockers

Beta-blockers such as propranolol, metoprolol, atenolol, bisoprolol, timolol, and nadolol are the first-line option for migraine prophylaxis and may be preferred in older adults with concomitant hypertension and coronary artery disease [72], but caution is advised as they may exacerbate congestive heart failure, bradycardia, asthma, diabetes, and depressive symptoms [73]. Lisinopril use can also be recommended for migraine prevention in individuals over 60 years of age, as no increase in side effects has been reported. Candesartan is also recommended for migraine prophylaxis, but data on its efficacy and safety is limited in older adults [32,72,74].

#### 9.2.2. Antiepileptics

Antiepileptic drugs, such as topiramate and sodium valproate, have demonstrated efficacy in migraine prophylaxis and are also used in older adults [75]. However, they should be carefully evaluated due to tolerability and safety concerns, such as cognitive and balance disorders, paresthesia, falls, and hepatic/renal effects [72,75].

#### 9.2.3. Antidepressants

Amitriptyline, a tricyclic antidepressant, has demonstrated efficacy in migraine prophylaxis compared with a placebo, although its use is often limited by poor tolerability [76]. Cognitive impairment, drowsiness, cardiac conduction problems, and increased side effects due to high plasma concentration limit its use in geriatric migraine patients [72,77]. Venlafaxine can be considered a good option as it has fewer side effects compared to amitriptyline [72,78].

#### 9.2.4. OnabotulinumtoxinA

OnabotulinumtoxinA has been used in many studies with the Phase III REsearch Evaluating Migraine Prophylaxis Therapy (PREEMPT) protocol in the treatment of chronic migraine, achieving a 50% response rate, and is a Food and Drug Administration-approved treatment [79,80]. Although studies in the geriatric migraine population are limited, it is reported to be safe due to its lack of systemic effects and only local effects such as pain at the injection site and temporary muscle weakness [81].

#### 9.2.5. Anti-CGRP Monoclonal Antibodies or Their Receptor

Data suggest that Anti-CGRP Monoclonal Antibodies (mAbs) can be safely administered in migraine prophylaxis for individuals under 65 years of age [72]. Furthermore, erenumab, galcanezumab, and fremanezumab have been shown to exhibit a well-tolerated and safe profile in individuals over 60 years of age [82,83].

#### 9.2.6. Gepants

Data specific to older adults on the long-term safety and tolerability of ategepant are still limited, as most clinical studies have a limited number of participants >65 years of age [84]. For rimegepant, a single 75 mg dose shows similar plasma parameters in older and non-older adult groups, and no age-based dose adjustment is required for a single dose [66]. The most common side effects are mild nausea and urinary tract infection. Hypersensitivity reactions, including shortness of breath and rash, may occur several days after administration. In such a case, rimegepant should be discontinued. Furthermore, age has not been shown to have a significant effect on safety signals, and tolerability has been reported in the adult population in long-term open-label studies; however, data are limited in very elderly (>75–80) patients and those with multiple comorbidities [85].

#### 9.2.7. Flunarizine

Flunarizine is a calcium channel blocker; however, adverse effects such as extrapyramidal symptoms (Parkinsonism), tremor, sedation, weight gain, and depression have been reported more frequently in older adults [86,87]. For these reasons, clinical recommendations suggest that Flunarizine should not be preferred in older adults in the first steps or should be used very cautiously, with regular neurological/psychiatric monitoring and evaluation of alternative prophylactic agents [5].

## 10. Non-Pharmacological and Multidisciplinary Management

Migraine management in older adults may incorporate non-pharmacological approaches alongside pharmacological treatment, depending on patient and clinician preference or when pharmacological options are contraindicated or poorly tolerated. Non-pharmacological interventions should be selected with an emphasis on safety, tolerability, and feasibility in older adults [5].

### 10.1. Physical Therapy and Rehabilitation Approaches

Physical therapy and rehabilitation approaches include exercises (strengthening, stretching, aerobics, yoga, etc.), manual therapies (mobilization, manipulation, osteopathy, etc.), soft tissue techniques (massage, myofascial applications), and electrotherapy approaches for pain. Data on physical therapy and rehabilitation approaches applied directly to older adults are insufficient. However, if applied in clinics, general geriatric care considerations should be taken into account. Age-related reductions in muscle mass and body water, together with an increase in fat mass, may affect drug distribution and increase treatment-related risks in older adults [88]. Therefore, in geriatric migraine patients, non-pharmacological strategies such as strengthening exercises to preserve muscle mass, evidence-based aerobic exercise to reduce migraine symptoms [89] and the addition of strengthening, stretching, and stabilization exercises in the presence of neck pain [90,91] may provide preventive benefits while minimizing systemic risk and targeting multiple physiological systems. Although data specifically addressing individuals aged ≥65 years remain limited, exercise programs initiated at mild to moderate intensity for 10–20 min are generally recommended, as they reduce the risk of falls, fatigue, and CV complications, while providing benefits for both muscle mass and migraine symptoms [91].

However, programs should be tailored to individuals, taking into account age-related osteoarthritic changes in the cervical region and balance impairment [92]. Temporary local pain and fatigue and tissue tenderness may occur. Although serious adverse effects are reported to be absent or rare in the literature, high-speed applications should be avoided, and comorbidities and contraindications should be thoroughly investigated before treatment [93]. Table 3 shows the main physical and rehabilitation approaches in older adults with migraine.

### 10.2. Psychological and Behavioral Therapies and Patient Education

Psychological and behavioral therapies, including relaxation techniques, stress management, and mindfulness-based approaches, represent key components of multidisciplinary care in older adults with migraine [94]. These interventions typically involve strategies such as relaxation training with biofeedback, stress reduction, desensitization to stress-related triggers, and the development of adaptive coping skills to modify maladaptive behavioral patterns [95]. Although data in older populations remain limited, these approaches are particularly relevant in addressing psychosocial factors that contribute to migraine burden, especially given the frequent co-occurrence of migraine with sleep disturbances, anxiety, and depression [96,97,98]. Furthermore, these interventions are especially valuable in older adults, who are more vulnerable to polypharmacy and medication-related adverse effects, as they provide patient-centered coping strategies with a low risk of systemic side effects [96].

## 11. Geriatric Migraine Is Grounded in Geroscience

From a geroscience perspective, migraine in older adults should not be interpreted solely as a neurological disorder, but as a condition emerging from the interaction between biological aging processes and disease-specific mechanisms [3]. Core hallmarks of aging, including chronic low-grade inflammation (inflammaging), vascular dysfunction, mitochondrial impairment, and altered neuroimmune signaling, may directly influence migraine susceptibility, phenotype, and treatment response [3]. This framework provides an added interpretive layer beyond traditional geriatric neurology, allowing migraine to be understood as part of a broader systemic aging process [99].

With aging, the classical presentation of migraine frequently fades, and the condition may manifest with atypical features, including non-throbbing pain or aura without headache [4,9]. This clinical ambiguity can overlap with serious age-related conditions such as TIA or giant cell arteritis, making differential diagnosis particularly challenging and requiring expertise that integrates geroscience principles [99].

A further unmet need lies in the management of multimorbidity. In older patients, migraine frequently coexists with conditions such as hypertension, diabetes, obesity, and cardiovascular disease, increasing clinical complexity and limiting the effectiveness of single-disease approaches [100,101].

The most critical gap, however, remains in treatment. Older adults have been largely excluded from pivotal clinical trials, resulting in limited evidence on the safety and efficacy of available therapies in this population. Age-related physiological changes, together with polypharmacy and comorbidities, increase the risk of drug–drug and drug–disease interactions [4]. Consequently, commonly used treatments such as triptans may be contraindicated, while traditional preventives are often poorly tolerated [13,14].

Novel therapies, including CGRP-targeted agents, offer promising alternatives, but robust data in older populations, particularly regarding long-term cardiovascular safety, are still lacking [13,14]. In addition, the potential relationship between migraine and cognitive decline remains an unresolved issue, warranting further investigation.

Future research must prioritize the inclusion of older adults in clinical trials and adopt a multidimensional approach that reflects the complexity of geriatric patients, addressing multimorbidity, polypharmacy, and long-term outcomes within a geroscience framework. In this context, the integration of artificial intelligence and advanced data-driven approaches may support more accurate phenotyping, risk stratification, and the development of personalized therapeutic strategies, ultimately improving clinical decision-making and patient outcomes [102]. The levels of evidence are summarized in Table 4.

## 12. Limitations

This review has some limitations. First, although priority was given to studies involving older adults, part of the evidence is derived from mixed-age populations or extrapolated from the general adult migraine literature. Second, randomized controlled trial data specifically addressing migraine management in older adults remain limited. Third, some mechanistic considerations are based on indirect evidence and should be interpreted as hypothesis-generating. Finally, as a narrative review conducted in accordance with the SANRA guidelines, this work does not follow a systematic methodology and may therefore be subject to selection bias. Nevertheless, emphasis was placed on clinically relevant and methodologically sound studies to provide a comprehensive and balanced overview of the topic.

## 13. Geriatric Migraine Embedded in SDG Project

In summary, the field of geriatric migraine is at a critical juncture. Geriatric migraine can be more appropriately conceptualized within the SDG framework as a multidimensional health challenge that spans clinical care, public health, and health system organization. Rather than representing a purely neurological condition, it reflects the intersection of aging-related vulnerability, chronic disease burden, and systemic healthcare gaps [3]. In particular, its impact on disability and quality of life aligns with SDG 3 (Good Health and Well-being), while the underrepresentation of older adults in clinical research reflects inequalities addressed by SDG 10 (Reduced Inequalities). Furthermore, the challenges of polypharmacy and safe medication use in this population are closely linked to SDG 12 (Responsible Consumption and Production) [3].

We now possess robust epidemiological evidence and growing societal awareness of the burden of migraine in older adults [3]. However, despite the availability of promising therapeutic options, there remains a critical need for high-quality data on safety and efficacy specifically in elderly populations [13,14,103]. At the same time, global frameworks linking the World Health Organization with the SDGs already provide a strategic foundation for addressing this challenge [1,3].

What is currently lacking is the operational knowledge required to translate these frameworks into safe, effective, and context-specific clinical practice [103]. Bridging this gap will require a coordinated research effort aimed at moving from extrapolated evidence toward truly evidence-based care tailored to older adults.

In this context, rather than relying solely on established global burden estimates [6], the next decade should focus on pragmatically identifying and addressing real-world clinical and healthcare system challenges associated with migraine in aging populations. Integrating these efforts within the framework of the 17 SDGs will be essential to align clinical innovation with global health priorities and to ensure sustainable, equitable care for this growing and vulnerable population [3].

## Figures and Tables

**Table 1:** Acute treatments and their safety in older adults with migraine [5,71].

Drug Class	Agents	Clinical Role	Geriatric Considerations	Key Risks/Cautions
Simple analgesics	Paracetamol	First-line in mild attacks	Prefer lowest effective dose; monitor liver function	Hepatotoxicity in cases of frailty/polypharmacy
NSAIDs	Ibuprofen, naproxen, diclofenac	Second-line	Use short-term only; assess GI, renal, and CV risk	GI bleeding, renal failure, CV events
Triptans	Sumatriptan, rizatriptan, etc.	Moderate–severe attacks	Use only if low vascular risk; careful patient selection	Stroke risk, CAD, hypertension
Ditans	Lasmiditan	Alternative when triptans contraindicated	Avoid in cases of frailty due to CNS effects	Sedation, dizziness, falls
Gepants	Ubrogepant, rimegepant	Promising option	Limited geriatric data; evaluate drug interactions	CYP3A4 interactions
Opioids/barbiturates	—	Not recommended	Avoid due to poor efficacy and high risk	Dependence, sedation, delirium

**Table 2:** Preventive treatments and their safety in older adults with migraine [5].

Drug Class	Agents	Clinical Role	Geriatric Considerations	Key Risks/Cautions
Beta-blockers	Propranolol (non-selective), Metoprolol, Atenolol, Bisoprolol (cardioselective)	First-line in selected patients	Prefer cardioselective in cases of CV comorbidity; monitor BP/HR	Bradycardia, hypotension, fatigue
ARBs	Candesartan	Alternative option	Useful in cases of hypertension	Hypotension, falls
Antiepileptics	Topiramate	Effective but limited use	Avoid in cases of cognitive impairment	Cognitive decline, falls
Antiepileptics	Valproate	Rarely used	Avoid in cases of frailty	Hepatic, metabolic effects
Antidepressants	Amitriptyline	Limited use	Avoid in elderly individuals (anticholinergic burden)	Cognitive impairment, arrhythmias
Antidepressants	Venlafaxine	Better tolerated	Consider in cases of comorbid depression	Hypertension
CGRP mAbs	Erenumab, fremanezumab, galcanezumab	Promising	Favorable safety but limited data for elderly population	Long-term CV safety unknown
Gepants	Atogepant, rimegepant	Emerging option	Monitor interactions	Hepatic, CYP3A4
OnabotulinumtoxinA	—	Chronic migraine	Preferred in cases of frailty (low systemic exposure)	Local pain, muscle weakness
Flunarizine	—	Last-line	Avoid in elderly individuals when possible	Parkinsonism, depression

**Table 3:** Physical therapy and rehabilitation approaches in older adults with migraine *.

Approaches	Components	When to Consider (Clinical Indications)	Older Adult–Specific Considerations and Their Safety
Aerobic Exercise	Low-intensity walking, cycling, etc.	Reduction in migraine symptoms and improvement in overall physical fitness	Controlled and individualized application reduces the risk of exercise acting as a migraine trigger Attention should be paid to the risk of falling
Cervical Exercises	Strengthening, stretching, stabilization exercises	Coexisting neck pain, cervical dysfunction, or reduced cervical mobility	Individualization is essential due to cervical osteoarthritic changes and balance problems
Manual Therapy	Mobilization, manipulation, osteopathic techniques	Migraine accompanied by cervical musculoskeletal dysfunction or muscle tension	High-velocity techniques should be avoided; osteoporosis and balance impairment must be assessed
Soft Tissue Techniques	Massage, myofascial release	Local muscle tenderness, increased cervical muscle tone	Temporary local tenderness or fatigue may occur; serious adverse events are rare

* These approaches should be individualized according to patient-specific characteristics.

**Table 4:** Classification of evidence across manuscript sections.

Section	Type of Evidence	Source of Evidence	Interpretation
Epidemiology	Direct evidence	Studies including older adults and population-based cohorts	Robust evidence specific to older populations
Clinical Presentation and Diagnosis	Mostly direct, partially extrapolated	Studies in older adults and mixed-age cohorts	Generally reliable, with some extrapolation required
Comorbidities and Differential Diagnosis	Mixed evidence	Observational studies in older adults and broader adult populations	Moderate evidence, partially extrapolated
Quality of Life and Functional Impact	Mixed evidence	Population-based studies and mixed-age cohorts	Consistent findings, partly extrapolated
Disease Burden and Healthcare Utilization	Mixed evidence	Registry-based and population studies	Reliable trends, with some extrapolation
Pathophysiology	Indirect/hypothesis-generating	Experimental models and studies in younger populations	Mechanistic interpretation, limited age-specific data
Treatment and Safety	Largely extrapolated	Clinical trials in general adult populations	Requires cautious interpretation in older adults
Geroscience Framework	Hypothesis-generating	Aging biology and neuroimmune research	Conceptual framework requiring validation in migraine
SDG Framework	Conceptual/policy-based	Global health and public health frameworks	Interpretative and strategic perspective

## Data Availability

No new data were created or analyzed in this study.
